# A Performance-Based Incentives System for Village Health Workers in Kisoro, Uganda

**DOI:** 10.5334/aogh.2400

**Published:** 2019-03-21

**Authors:** Crystal Zheng, Sam Musominali, Gloria Fung Chaw, Gerald Paccione

**Affiliations:** 1 Tulane University, US; 2 Doctors for Global Health, UG; 3 Albert Einstein College of Medicine, US

## Abstract

**Background:**

Village health worker (VHW) programs in Uganda have achieved limited success, due in part to a reliance on volunteerism and a lack of standardized incentive mechanisms. However, how to best incentivize VHWs remains unclear. Doctors for Global Health developed a performance-based incentives (PBI) system to pay its VHWs in Kisoro, Uganda, based on performance of tasks or achievement of targets.

**Objectives:**

1. To describe the development of a PBI system used to compensate VHWs. 2. To report cost and health services delivery outcomes under a PBI system. 3. To provide qualitative analysis on the successes and challenges of PBI.

**Methods:**

Internal organization records from May 2016 to April 2017 were retrospectively reviewed. The results of descriptive and analytic statistics were reported. Qualitative analysis was performed by the authors.

**Findings:**

In one year, 42 VHWs performed 23,703 remunerable health actions, such as providing care of minor ailments and chronic disease. VHWs earned on average $237. The total cost to maintain the program was $29,844, or $0.72 per villager. There was 0% VHW attrition. Strengths of PBI included flexibility, accountability, higher VHW earnings, and improved monitoring and evaluation.

**Conclusions:**

PBI is a feasible and sustainable model of compensating VHWs. At a time where VHW programs are sorely needed to address limitations in healthcare resources, yet are facing challenges with workforce compensation, PBI may serve as a model for others in Uganda and around the world.

## Introduction

Uganda is a World Bank-classified, low-income country in Eastern Africa that faces significant challenges in health services delivery. Based on World Health Organization (WHO) data, the average life expectancy in Uganda is 62.3 years, ranking 154 out of 183 countries [1]. Infectious diseases account for the top four causes of mortality, and 343 women die for every 1,000 live births [2]. Seventy-five percent of Ugandans live in rural areas, where health outcomes are even worse and access to care more difficult [4]. A lack of financial and human resources presents a major barrier to improving the country’s health outcomes.

In light of these challenges, the Uganda Ministry of Health initiated the Village Health Teams (VHT) program in 2001. Village health worker (VHW) programs have been demonstrated to be successful in a variety of settings including Ethiopia, Nigeria, and Mali, and they are strongly endorsed by the WHO as a cost-effective health delivery strategy [3]. However, Uganda’s VHT program has had problems with both effectiveness and sustainability, and it has realized limited success, particularly when measured against the Millennium Development Goals. A 2015 national assessment of VHT identified many implementation weaknesses, including a reliance on volunteerism and lack of standardized incentive mechanisms. In response, the Ministry of Health created the Community Health Extension Worker (CHEW) program, which, among other objectives, aims to “strengthen the training, motivation and performance management of CHEWs.” To this end, the government will pay CHEWs a fixed salary as well as financial incentives based on performance evaluations [4].

How to best incentivize VHWs remains unclear. VHWs can receive monetary and non-monetary incentives, and monetary incentives can take many forms, such as salary, per diem, or performance based. USAID evaluations of multiple VHW programs found that monetary incentives can be motivating and result in higher retention, but they can also be problematic if they are not sufficient, paid irregularly, result in medical overtreatment, or undermine relationships with the community. However, data regarding the effect of monetary incentives on service delivery and health outcomes are lacking [5,6].

We describe our experience implementing a performance-based incentives (PBI) system, whereby VHWs are paid for performance of tasks or achievement of targets. We summarize the evolution of the program over time, report program outcomes from 2016–2017 in terms of VHW income and health services delivery, and provide qualitative analysis of the successes and challenges of PBI. As the Ugandan Ministry of Health has identified the lack of incentives as a key limitation of its prior VHT program, we believe that our account will have important implications for Uganda and beyond.

### Description of village health worker program and performance-based incentives

Kisoro district is in southwest Uganda, 200 kilometers away from Mbarara, the nearest major city. Its population of 287,000 includes 94% living in rural areas and 89% working as subsistence farmers. Only 17% of adults have completed primary school [7,8]. In January 2007, Kisoro District Hospital (KDH) initiated a VHW program with a mission to deliver broad primary care. The program is funded by Doctors for Global Health (a US-based NGO) and by partnerships with US medical schools that contribute funds to support global health opportunities for medical students. In this way we established a collaborative model of medical education that serves the sponsoring community and keeps the VHW program financially sustainable. Whereas many VHW programs are “vertical,” delivering one type of intervention, ours is a “horizontal” program in which VHWs deliver a comprehensive set of preventive, educational, and curative health actions. The program currently includes 53 VHWs and covers a population of roughly 50,000 across 50 villages.

The program’s payment system has evolved since its inception. Initially in 2007, VHWs were paid a monthly salary of approximately $15 in good faith for two full workdays per week. However, it was difficult to quantify how much work was actually accomplished. In October 2008, in an effort to more specifically define a set of actions at the family level, payment was switched to a per-household system. VHWs visited households and documented any health issues encountered, such as malnutrition and pregnancy. They were paid 300 Ugandan shillings (or $0.18) per household, up to 80 households per month. However, the low rate of clinical events and lack of accountability led to suspected forgery of data. As a result, the data recorded by VHWs during these “household visits” was also deemed unreliable.

In May 2010, the program switched to a PBI system that assigned a relative value to, and paid VHWs based on, certain measurable health delivery outcomes. VHWs received no base pay. Instead, they were compensated for performance of various activities. Stipend amounts for each activity were determined based on our assessment of its public health or clinical importance, incidence, and effort required of VHWs. The exact stipend scheme has gone through many iterations, and the current scheme is detailed in Table 1.

**Table 1:** Performance-based incentives stipend amounts.

Incentive item	Stipend	

	Ugandan shillings	US dollars

**Acute illness**

Doctor bag used correctly	1500	$0.44
**Identification of new patients**

Malnourished child	3000	$0.87
Treatable disability	3000	$0.87
Pregnancy identified and delivery plan developed	2000	$0.58
Chronic disease (hypertension, epilepsy, diabetes, heart failure, asthma, tuberculosis, HIV)	2000	$0.58
Mental illness	2000	$0.58
Domestic violence	1000	$0.29
Recent birth	800	$0.23
“Difficult home” (child abuse, immunization refusal, other family problems)	500	$0.15
**Referrals/follow up visits**

Malnourished child weighed and food delivered (malnutrition cycle)	5000	$1.46
Follow up of recent hospital discharge	1000	$0.29
Chronic disease encounter through “CDcom”	1000	$0.29
Family planning referral	1500	$0.44
Antenatal care referral	1000	$0.29
Cervical cancer screening referral	1000	$0.29
Immunization referral	1000	$0.29
High risk chronic disease follow up visit	200	$0.06
Home talk presentation	500	$0.15

Initially, remunerable activities consisted mainly of case identification, such as chronic disease, pregnancy, malnutrition, and poor sanitation. As VHWs gained experience, interventional activities such as use of doctor bags, care of chronic disease, and delivery of home talks were added. Each VHW was given a doctor bag filled with practical tools including first aid supplies, blood pressure cuff, mid-upper arm circumference strips, pregnancy tests, oral rehydration solution, acetaminophen, and amoxicillin. For each complaint, VHWs were required to correctly apply physician-created guidelines to determine a course of action. Patients with chronic diseases were cared for under the auspices of “Chronic Disease in the Community,” or CDcom, a program in which VHWs diagnose, monitor, and provide medications for those with chronic diseases. VHWs could also deliver “home talks,” standardized presentations on common health topics such as malnutrition, hypertension, and cervical cancer screening based on preidentified educational needs of each household. The number of paid home talks per month was tightly capped based on the quality of a VHW’s presentations as evaluated by a supervisor. Most VHWs did not complete training in delivering home talks until December 2016.

Furthermore, VHWs received monetary bonuses for completing advanced training, performing a census of their village every two to three years, and based on performance percentiles within the group. Training bonuses were awarded for completing certification in five domains: child wellness, women’s health, chronic disease, environmental health and sanitation, and acute illness. Supervisors visited each village twice a month to verify the VHWs’ claimed activities. The stipend data recorded by VHWs was summarized in a monthly report, which provided useful information to the program on VHW productivity and the health status of the community.

After one year of the PBI program’s implementation, Miller et al. performed a process evaluation and found that VHWs perceived the system to be more “fair” than the prior household visit-based system, which they realized was rife with fraud. However, despite an increase in average stipends, some complained that payments were too low and less predictable compared to the previous system. The evaluation also found that attrition was low, and VHWs with limited education were able to master the PBI scheme.

From 2012 to 2016, we implemented an insurance program with the hope that payments from villagers would sustain the VHW program and keep it financially independent from outside funding sources. We asked families to pay eight dollars per year in exchange for heavily subsidized transportation to the hospital. Additionally, many health services performed by VHWs were only available to villagers who joined. Regrettably, the program achieved only 10–20% membership within each village, limiting overall activity. In March 2016, the insurance program ended, and the VHW program was opened once again to all villagers.

## Methods

Data on VHW and village demographics, VHW income, health services delivery, attrition, and program costs were obtained by retrospective review of internal records during the 12 months of May 2016 to April 2017. We chose this timeframe to remove the confounding effects of the insurance program that ended in March 2016, and of the recruitment of a new cohort of VHWs in May 2017. Eighteen health activities are examined (Table 1). In reporting VHW income, “stipend” refers to the amount paid to a VHW for a single action. “Base pay” refers to the sum of a VHW’s stipends earned from all activities. “Real pay” refers to the amount paid to a VHW including bonuses. We used a fixed exchange rate of 1 US dollar to 3,429.94 Ugandan shillings, which was the median exchange rate on the first of each month during the timeframe studied [9], and rounded to the nearest dollar. ANOVA was used to compare outcomes between discrete variables, and linear regression was used to assess the relationship with continuous variables. Statistical analysis was performed using STATA 12.1. Qualitative assessment was performed by the authors based on personal experience. The authors were involved in the design, implementation, and day-to-day operations of PBI and the VHW program; thus, this analysis cannot be considered an independent evaluation. This study was approved by the Albert Einstein College of Medicine Institutional Review Board.

## Results

### 1. Who are our VHWs?

During the timeframe studied, our program consisted of 42 VHWs, 35 women and 7 men. VHWs ranged in age from 23 to 67 with a median age of 38. One hundred percent of men had a secondary level of education, compared to 44% of women (P < 0.01). Eleven VHWs had been trained in 2007 (cohort 1), 13 in 2009 (cohort 2), and 18 in 2014 (cohort 3); accordingly the cohorts had 9, 7, and 2 years of experience, respectively, at the beginning of the timeframe studied. The average distance from a VHW’s village to KDH was 10 kilometers (range 5–16.5, SD 2.88). The median VHW covered 195 households, ranging from 78 to 389. With an average of 5 persons per household based on our census data, the VHWs collectively covered 8,264 households and an estimated 41,320 persons.

### 2. How much does PBI cost?

The total cost to maintain the program for one year was $29,844, or $711 per VHW and $0.72 per villager. The sum of real pay for 42 VHWs was $9,960. Administration accounted for 4% of total costs while other costs went directly into providing services for the community. Detailed costs are itemized in Table 2.

**Table 2:** Program costs over one year.

Item		Cost ($)

Administration		$1,336
	*Percentage of administrator’s salary according to percentage of time dedicated to the VHW program*	
Transportation		$1,473
	*Up-front cost of 10 motorcycles divided by an expected lifetime of 10 years, motorcycle maintenance, fuel, and transportation vouchers*	
Supervisor stipend		$8,572
	*Paid per diem at a level commensurate with supervisor experience*	
Training		$3,444
	*Includes cost of initial training divided over 7 years, the average length of VHW involvement, and continued medical education*	
CDcom medications		$3,888
Doctor bags		$1,170
	*Includes the upfront cost of $100 per bag, divided by an expected lifetime of 10 years, and the cost to replenish supplies for one year*	
VHW stipends		$9,960
**Total cost**		$29,844

### 3. VHW income

**Figure 1:**
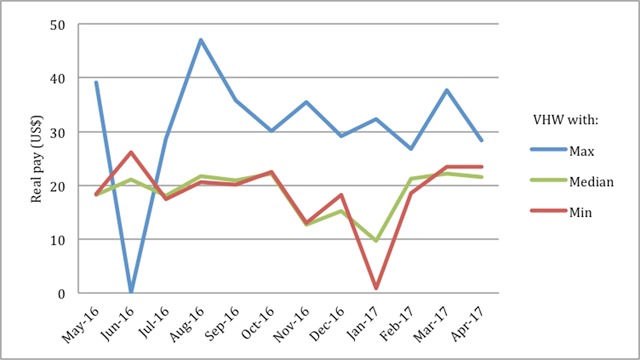
Monthly real pay for VHWs with the maximum, median, and minimum earnings over 12 month period.

The average VHW earned $214 from stipend activities in one year. Once bonuses were calculated, the average VHW earned $237 in real pay, providing an 11% increase in income. However, there was wide variation in income among VHWs, with the lowest performer earning a real pay of $129 and the highest performer earning $370 (SD $54) (Figure 1). There was no significant difference or correlation in real pay with sex, age, years of experience, level of education, number of households, or distance to KDH.

**Figure 2:**
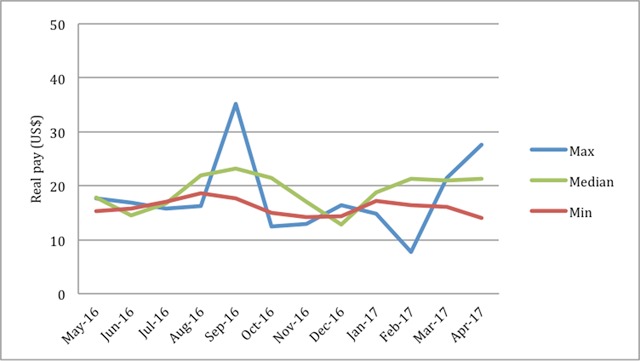
Monthly real pay for VHWs with the maximum, median, and minimum month-to-month ranges over 12 month period.

Some VHWs earned a relatively consistent amount from month-to-month while others earned a more variable amount. Removing months where VHWs earned no income, the average range in monthly real pay for a single VHW was $13, with a minimum of $5 and a maximum of $27 (Figure 2).

### 4. Attrition

In May 2016 our program consisted of 42 VHWs. In April 2016 all 42 original VHWs continued to work with us, reflecting a 0% attrition rate over one year.

### 5. Health impact

As a group, the VHWs identified 1,634 recent births, 665 difficult homes, 367 homes with domestic violence, 332 malnourished children, 186 cases of asthma, 168 cases of mental illness, 175 cases of hypertension, 83 cases of alcohol abuse, 54 cases of HIV, 35 cases of epilepsy, 28 cases of treatable disability, 19 cases of TB, 15 cases of diabetes, and 8 cases of heart failure.

In one year, the median performing VHW used a doctor bag to treat acute illness 182 times, cared for CDcom patients 164 times, identified or followed up on 70 chronic disease patients at home, followed up on 18 recent hospital discharges, referred 39 patients for immunization, 24 for antenatal care, 9 for cervical cancer screening, and 4 for family planning, and gave 11 home talks.

**Figure 3:**
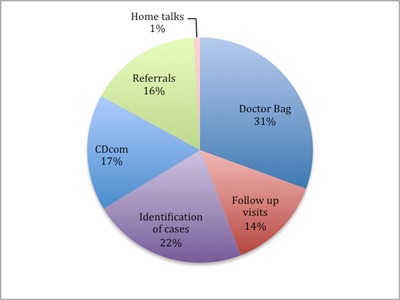
Health services delivered by category.

Overall, the most common activity was doctor bag usage. In total the VHWs used doctor bags 7,254 times, accounting for 35% of total stipends. Other top activities were CDcom (3,951), high-risk chronic disease follow-up visits (1,814), and antenatal referrals (1,814). The least performed activities were identification of domestic violence (367), malnourished children (332), mental illness (168), disability (28), and home talks (205) (Figure 3). Possible explanations for the low numbers of these activities will be reviewed in the discussion.

**Figure 4:**
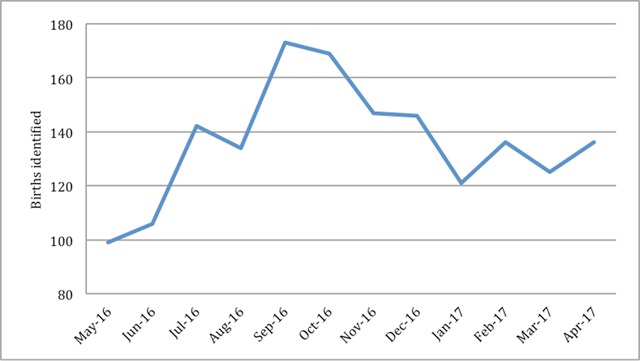
Total number of recent births identified by VHWs by month.

**Figure 5:**
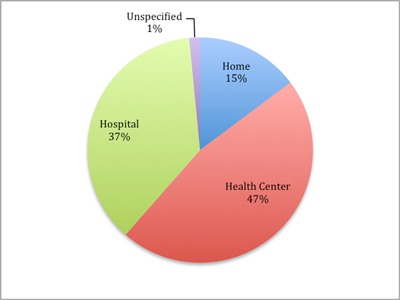
Delivery location of recent births identified by VHWs.

Data generated by VHWs identified some epidemiologic trends. For example, the number of malnourished children identified was highest in October, correlating with the period before the harvest. Similarly, identification of recent births peaked in September, correlating with migrant worker husbands returning home during Christmas (Figure 4). Of pregnant women identified by VHWs, 84% delivered in a health care setting, either a health center or hospital (Figure 5).

### 6. Homogeneity and heterogeneity of VHW Behavior

The distribution of health activities performed by VHWs as a group was relatively homogeneous. Doctor bag use was the most common activity for 38 VHWs, and CDcom was among the top two items for 30 VHWs. Although not statistically significant, women tended to perform more family planning referrals than men (P = 0.07), with an average of 16 compared to 10, despite there being no difference in the number of pregnancies or recent births identified. Identification of disability was the least performed item, ranking in the bottom five for 41 VHWs, with 25 performing none at all. Other activities consistently in the bottom five were home talks for 31 VHWs and identification of mental illness for 30 VHWs.

Given the dominance of the top two activities, which was in part by design, individual VHW behavior is better reflected by examining the remaining 16 activities. Immunization referral and chronic disease follow-up as a percentage of a VHW’s total activity had high variation, which we define as a standard deviation greater than 5%. Immunization referral accounted for between 0–39% (SD 10%) of a VHW’s total activities, and chronic disease follow-up for 1–25% (SD 6%). The remaining 14 activities were performed consistently by all 42 VHWs. Intra-VHW variability (i.e., variation in a single VHW’s activity from month to month) also provides insight into VHW behavior. After removing doctor bag and CDcom, most VHWs had 11 activities that were performed consistently and five activities that varied from month to month. The activities with the highest amount of month-to-month variation among all 42 VHWs were recent birth, recent pregnancy, and chronic disease follow-up.

Certain activities had statistically significant correlations with one another. For example, the number of malnourished children identified or treated by a VHW positively correlated with the number of recent births identified. VHWs that followed up on more hospital discharges also identified higher numbers of chronic disease. who that identified more difficult homes also identified more domestic violence and mental illness.

### 7. Response to incentives

**Figure 6:**
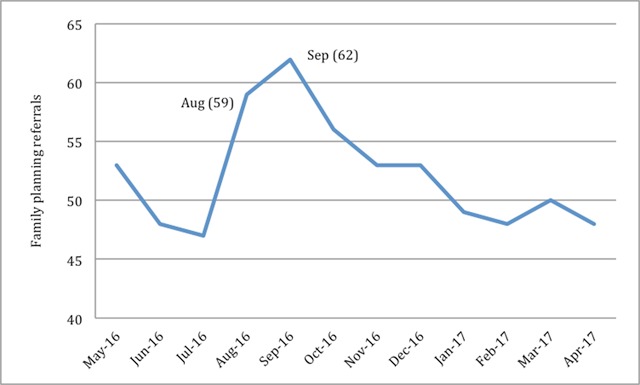
Total number of family planning referrals performed by VHWs by month (incentive months labeled).

Another attribute of VHW behavior is how he or she responds to incentives. Our program implemented an “incentive of the month” initiative, which increased a given month’s stipend for certain activities. Most incentives were epidemiologically based; for example, the stipend for family planning referrals was tripled in August and September to encourage VHWs to discuss family planning with women during months with peak birth rates. Accordingly, family planning referrals peaked in August and September (Figure 6).

## Discussion

Our program’s PBI system is now entering its eighth year. Our experience shows that using PBI to pay VHWs is a feasible, sustainable, and beneficial model. Of course, we also experienced challenges that led to innovative adaptations throughout its implementation. In this section, we discuss the data presented above, and also provide qualitative analysis of the strengths and weaknesses of PBI based on our personal experiences.

### Successes

1. Health services delivery: PBI incentivized the program’s 42 VHWs to perform a total of 23,703 health actions in one year. Every day, an average of 20 villagers were treated for minor ailments by VHWs using doctor bags. VHWs visited patients who were recently discharged from the hospital 772 times in a region with no other mechanism for post-discharge follow up. Nearly 4,000 chronic disease encounters were made, averting the need for those in remote villages to spend time and money to travel to the hospital. The 1,634 births identified by VHWs accounted for 77% of the predicted number of births in the covered villages (C. Khan, personal communication, March 30, 2018). VHW training emphasized working with women to create a safe birth plan, and 84% of new mothers visited by VHWs delivered in a healthcare setting, compared to 70% regionally [10]. These are just some examples of the work accomplished by the program’s VHWs. With a catchment area population of over 40,000, VHW services are provided at an annual cost of $0.72 per person.

Commonly performed activities can partly be explained by program design. VHWs received significant training in use of doctor bags, and doctor bags can be applied to multiple different clinical scenarios. Chronic disease was a large focus of our program, with establishment of a structured system for management, the ability to increase stipends through targeted certification, and emphasis through incentive of the month. Certain activities were performed infrequently, namely identification of domestic violence, malnourished children, mental illness, disability, and delivery of home talks. It is possible that individuals experiencing domestic violence or mental illness may not approach VHWs due to cultural stigma. VHWs were limited in the number of home talks they could perform while training was in process, but we anticipate home talks will be a popular activity once certification is complete. For other infrequently performed activities, it is unclear whether these numbers reflect low prevalence in the community or insufficient monetary incentives to perform these tasks.

2. VHW income: Under PBI, VHWs earned more on average than under the previous household system. The household system paid VHWs a maximum of $16.12 per month, while the monthly average income under PBI was $19.76, a 23% increase. To place VHW income in perspective, the local rate to hire a laborer in the fields is approximately two dollars for a full day’s work. Almost all Kisoro villagers, including VHWs, generate income from farming. Assuming VHWs spend roughly 16 hours, or the equivalent of two days per week on VHW work, the average daily rate for VHW work is $2.28, which is meant to supplement, not replace, income from traditional field labor. However, in informal interviews with VHWs, it appears that most actually work only eight hours per week. On a daily basis, the rate for VHW work more than compensates for the opportunity cost of not working in the fields.

3. Workforce: An initial evaluation in 2011 after one year of implementation of PBI found that PBI was well accepted by VHWs. Our data from 2016–2017 continues to support this. We experienced a 0% attrition rate over one year, in comparison to annual attrition rates reported in the literature of 3–77% [5]. Although the low attrition rate during 2016–2017, as well as in other years not formally studied here, in part reflected VHW satisfaction, we believe another key factor was the lack of other economic opportunities in the area. Data gathered by PBI also allowed us to identify relatively inactive VHWs and encourage them to either improve performance or limit their role in the program, thereby enhancing the quality of our workforce. Since the time of our study, we continue to have community interest in joining the program and have been successful in recruiting a new cohort of VHWs.

4. Incentives: A fundamental assumption of PBI is that VHWs respond to incentives and therefore are more productive. VHWs performed more family planning referrals in the months where the stipend amount was increased, supporting a relationship (but not causation) between incentives and productivity. More likely, the increase in family planning referrals was due to a combination of increased incentives and increased seasonal demand, illustrating how we designed incentive of the month using the epidemiology and incentives together to maximize impact on the community.

In addition to direct financial incentives, we structured the execution of PBI to emphasize the concept of pay for performance. Stipend sheets standardized services and provided VHWs with guidance on the pursuit of specific health actions. Payment intervals were changed from quarterly to monthly for immediate reward of effort. High-performing VHWs received bonus multipliers based on earnings tercile, using the assumption that base pay was a proxy for activity level and involvement. Conversely, underperformers who earned the least base pay were identified and appropriate counseling triggered.

5. VHW behavior: In general, VHWs were homogeneous in the activities they performed. Variation among VHWs most likely reflects individual preferences, and is unlikely to reflect a true variation in prevalence between villages. Correlations between certain activities perhaps correspond to clusters of interest in pediatrics, chronic disease, or mental illness. Identification of recent births and pregnancies had high intra-VHW variation from month to month, which likely reflects the seasonality of birthrates. Otherwise, individual VHWs tended to perform the same activities each month.

Women tended to perform more family planning referrals than men, although this finding was not statistically significant. Hypothesized explanations could include the fact that women in the village may be more comfortable discussing family planning with other women, or that women VHWs are more attuned to the need for family planning. With this information, our program could increase awareness among male VHWs to encourage family planning referrals. Although cultural barriers exist, male VHWs could have a large impact since; for better or for worse, a husband’s buy-in is a large factor in a woman’s decision.

6. Accountability: Supervision is integral to any VHW program to ensure accountability. As opposed to a fixed payment model, PBI requires supervisors to verify specific actions. In our program, supervisors visit villages twice a month and confirm at least 70% of a VHW’s reported work. To keep supervision cost effective, supervisors only visit a VHW if at least eight activities have been reported. Although there is always the possibility of fraud even with supervision—VHWs directing supervisors away from certain homes, for example—this system greatly minimizes it.

Additionally, supervisors bring a higher level of expertise and legitimize the VHW in the eyes of the community. As a continuing education opportunity, supervisors use the time spent walking between houses to review monthly “Education Scripts” that cover a long-term curriculum. We have also identified high-performing VHWs and invited them to act as peer supervisors, giving these individuals opportunities for career advancement, economic earnings, and a sense of empowerment. We envision peer supervision will be a future direction of growth as the program expands in order to cost-effectively fill a need in a region with a short supply of health professionals.

7. Data gathering: Under a fixed-payment model, we had no data on activities performed by VHWs. Under a household model with little supervision, the data generated was unreliable. One benefit of PBI is the automatic generation of useful data. When VHWs record their activity for payment purposes, that data provides information on VHW productivity and community health status. This allows us to better identify community needs and assess our impact on the community. Additionally, because VHW self-reporting is verified by supervision, we are able to assure the quality of the data.

VHWs were able to capture over three-quarters of predicted births and a large number of other medical conditions. Knowledge of disease prevalence and temporal trends allow us to tailor and improve our services. For example, knowledge of peak months for malnutrition and recent births could prompt VHWs to be vigilant for these conditions. Additionally, we can track data, such as the proportion of women delivering in a health care setting, as a measure of our impact over time.

8. Flexibility: Based on program needs, which activities to remunerate and how much is easily adjusted. Our stipend scheme has undergone revisions every one to two years. For example, CDcom was added when we found an increased need in the community. Identification of latrines was an early item that was determined to be of low impact and removed. As VHWs gained more experience, activities requiring higher levels of training, such as home talks, were added.

PBI also provides flexibility for the VHW. While some VHWs earned a stable amount every month, others had large month-to-month variation. Some VHWs favor certain activities over others. This variation in income and activities reflects the fact that PBI allows VHWs the flexibility to choose when, how much, and what kind of work to perform.

9. Training: We incorporated incentives into PBI to encourage VHWs to further their training. VHWs received a stipend for attending training sessions and were encouraged to attain certification in five specific domains. VHWs received a permanent 10% increase in pay per certification, and an additional 50% percent increase for completing *all* certifications. The training bonuses were attractive to VHWs because they provided a stable, predictable supplement to short term bonuses from other means.

10. Non-monetary incentives: Although not the focus of this paper, we briefly describe the non-monetary incentives offered to our VHWs. As introduced previously, we provided VHWs with a “doctor bag” filled with basic diagnostic tools and symptomatic treatments. The total value of a complete doctor bag and one year of supplies was US$100. There is some concern in the VHW literature that performance-based or fee-for-service incentives may result in over treatment. In our program, VHWs received payment only for appropriate use of the doctor bag. As an example, a VHW who, per guidelines, incorrectly gives a coughing patient amoxicillin without other danger signs such as fever or tachypnea would not receive compensation. Other intangible non-monetary incentives are a sense of status in the community, self-empowerment from acquired knowledge and skills, and the opportunity for career advancement.

## Challenges

The initial evaluation in 2011 found that some VHWs were concerned about unpredictable income. Our data show that there is a wide variation in income between VHWs, and that there is also a range of month-to-month income generated by a single VHW. If the variation in income is due to performing the same amount of work but encountering fewer paid activities, the VHWs’ concerns would be valid. However, in that case we would expect general trends that affect all VHWs, which was not borne out by the data. More likely, the variation is due to VHWs’ own choice of work hours.

Another concern frequently brought up by VHWs is that they are punished for performing their job well; for example, a VHW that eliminates malnutrition in his or her village would not be able to continue claiming that activity. Though this fact may be valid, we do not believe it will influence VHW activity or income substantially. Our program emphasizes the concept of “horizontal care” and trains our VHWs to provide many services across multiple domains rather than one specialized service, and new activities are frequently added into the VHW’s repertoire.

At a program level, we needed to address the inherent cost unpredictability of PBI. Compared to a fixed payment model, the cost to pay VHWs in a PBI model varies depending on their productivity. In the event that VHW payments exceeded our budget, we established a system in which stipend amounts for each activity would be changed to be reported as a range. Each VHW would receive a proportional payment decrease within the listed range. Fortunately, we have never needed to implement this backup strategy.

### Limitations

Although we have many more years of data, we limited the study to these 12 months in order to remove the confounding effects of other program aspects. We lack data on actual VHW activity from prior payment methods and therefore cannot make quantitative comparisons between PBI and other models. We point out that the lack of data from prior models actually highlights the advantage of PBI in data gathering. Prevalence of medical conditions in participating villages is not fully captured in the data generated by PBI, because most cases are detected through other methods. Our data cannot be generalized, because our VHWs represent only 42 villages out of over 400 in Kisoro District, and it is not a random sample of villages. Though we present data showing the impact of PBI on health services delivery, we lack data showing an impact on health outcomes, which could be an area of future study. Finally, the qualitative assessment in this study is based off the authors’ personal experience and is therefore subject to the biases inherent in anecdotal evidence. It also does not reflect the experience of other people involved with VHW program, such as the VHWs themselves.

## Conclusion

Performance-based incentives is a feasible model of compensating village health workers, which we believe enables beneficial health services delivery. PBI is a sustainable model that we have used for seven years. However, our system was not created in its final form at once, but rather required years of fine-tuning. Major advantages of PBI are its retention, accountability, data gathering, and flexibility. PBI has worked for our specific context, but other VHW programs that wish to implement PBI will need to adjust the system to their unique needs. At a time where VHW programs are sorely needed to address limitations in healthcare resources yet are facing challenges with workforce compensation, our experience in Kisoro may serve as a model for others in Uganda and around the world.

## Data Accessibility Statement

All authors had access to the data and a role in writing the manuscript.
